# Infantile Diarrhœa

**Published:** 1897-08

**Authors:** 


### INFANTILE DIARRHEA.

A. F. Plicque (*La Presse Medicale*, July 1, 1896).—The author recognizes four varieties of diarrhea: (1) Simple diarhea, caused by cold or by defects in alimentation. This should be promptly treated by regulation of the diet, and by a purgative, such as castor oil with orange juice, or sodium sulfate in sugar water. Should further medication be required, injections of starch water with from one-half to a drop of laudanum may be employed, or the internal administration of bismuth or astringents. Persistent diarrhea must be treated by opium. (2) Bilious diarrhea, which may be discriminated from green bacillary diarrhea, by the addition of a drop of nitric acid to the stains made by the fecal matter, when the characteristic violet-rose coloration will be seen. Alkaline treatment is all important; sodium bicarbonate is given at the rate of fifteen grains in twenty-four hours for each two and one-half pounds of the child's weight, but the daily dose should never exceed more than one and one-half drams of the alkali. Biliary diarrhea due to tuberculois of the liver will resist all treatment. (3) Green bacillary diarrhea may be acute and cause death in one or two days, or becoming chronic, may prove fatal from athrepsia. In addition to general hygienic measures we should give lactic acid. (4) Cholera infantum may occur during the course of either of the varieties of diarrhea alluded to. Its two special symptoms are peripheral algidity with cyanosis of the extremities, with, perhaps, rectal elevation of temperature and collapse with complete inertia, which is occasionally interrupted by subsultus and respiratory efforts.

Feeding should be insisted upon in spite of the vomiting; chicken broth being often better tolerated than milk. Milk with a few drops of brandy should be given by teaspoonfuls. The medicinal treatment consists of lactic acid, hydrochloric acid, and laudanum. Internal stimulants, as champagne, coffee, etc., or external ones, as hot mustard baths and injections of ether, should be employed.—*Philadelphia Polyclinic.*

